# Supplementary material for: Machine learning-based models to predict the conversion of normal blood pressure to hypertension within 5-year follow-up
Source: PLoS One. 2024 Mar 14;19(3):e0300201. doi: 10.1371/journal.pone.0300201 (PMC10939282; doi:10.1371/journal.pone.0300201)
Supplement: S1 Table — (DOCX) [file pone.0300201.s001.docx]

**Supplementary Table 1**. All characteristics and clinical features of participants

| **Non-laboratory variables** | |
| --- | --- |
| Ethnicity | History of Back Stiffness |
| Age | History of Oral Aphthous |
| Marital status | History of Genital Aphthous |
| Education years | History of Rheumatoid Arthritis |
| History of Diabetes | Past Medical History of Surgery |
| History of Hypertension | Past Medical History of Hospitalization |
| History of Myocardial Infarction | Past Medical History of Transfusion |
| History of Stroke | Having job |
| History of Renal Failure | Sleep Duration |
| History of Fatty Liver | Diastolic Blood pressure |
| History of Hepatitis B | Systolic Blood pressure |
| History of Hepatitis C | Pulse Rate |
| History of Chronic Lung Disease | Waist Circumference |
| History of Thyroid Disease | Hip Circumference |
| History of Kidney Stone | Wrist Circumference |
| History of Gallstone | Waist-to-hip ratio |
| History of Rheumatic Disease | Waist-to-height ratio |
| History of Skin Cancer | Smoking |
| History of Stomach Cancer | Use Alcohol |
| History of Colorectal Cancer | Family History of Hypertension |
| History of Bladder Cancer | Family History of Cardiovascular Diseases |
| History of Esophagus Cancer | Family History of MI |
| History of Lung Cancer | Family History of Stroke |
| History of Brain or CNS Cancer | Family History of Stomach Cancer |
| History of Epilepsy | Family History of Colorectal Cancer |
| History of Chronic Headaches | Family History of Skin Cancer |
| History of Depression | Family History of Bladder Cancer |
| History of Psychiatric Disorder | Family History of Lung Cancer |
| History of Laryngeal Cancer | Family History of Brain or CNS Cancer |
| History of Tongue Cancer | Family History of Alzheimer |
| History of Lupus | Family History of Pelvic Femoral Fracture |
| History of Multiple Sclerosis | Family History of Laryngeal Cancer |
| History of Cardiovascular Diseases | Family History of Tongue Cancer |
| History of Sternum Irritation | Family History of Lupus |
| History of Urine Color Changes | Family History of Multiple Sclerosis |
| History of Heartburn | Physical Activity |
| History of Food Regurgitation | Socioeconomic Status |
| History of GERD | Total Lipid Fat Intake |
| History of Blood In Stool | Total Carbohydrate Intake |
| History of Weight Loss | Total Fiber Intake |
| History of Jaundice | Calcium Intake |
| History of Shortness of Breath | Cholesterol Intake |
| History of Gait Problem | Total Trans Fatty Acid Intake |
| History of Faint | Iron Intake |
| History of Visual Impairment | Sodium Intake |
| History of Muscle Weakness | Caffeine Intake |
| History of Movement Disorder | Grain Product Consumption |
| History of Numbness | Nut Consumption |
| History of Recurring Headaches | Meat Consumption |
| History of Dizziness | Dairy Product Consumption |
| History of Tinnitus | Vegetables Consumption |
| History of Five Year Fracture | Fruits Consumption |
| History of Fracture Ever | Sugar Products Consumption |
| History of Hip Femoral Fracture | Salt Consumption |
| History of Osteoporosis |  |
| History of Back Pain |  |
| History of Joint pain |  |
| History of Joint Stiffness |  |
| **Laboratory variables** | |
| WBC | Gamma-glutamyl transferase (GGT) |
| RBC | Urine Specific Gravity |
| Hemoglobin | Urine Nitrite |
| Hematocrit | Urine Bilirubin |
| MCV | Urine Urobilinogen |
| MCH | Urine Protein |
| MCHC | Urine Glucose |
| PLT | Urine Blood |
| BUN level | Urine Crystal |
| Serum Creatinine Level | Urine Ketones |
| Triglyceride Level | Urine Ascorbic Acid |
| Cholesterol Level | RDW of Blood Test |
| Glomerular Filtration Rate |  |
| Aspartate Aminotransferase (AST) |  |
| Alanine Transaminase (ALT) |  |
| Alkaline Phosphatase |  |
| HDL-C |  |
