# Supplementary material for: Machine learning-based models to predict the conversion of normal blood pressure to hypertension within 5-year follow-up
Source: PLoS One. 2024 Mar 14;19(3):e0300201. doi: 10.1371/journal.pone.0300201 (PMC10939282; doi:10.1371/journal.pone.0300201)
Supplement: S2 Table — #Abbreviations, LR; Logistic Regression, SVM; Support Vector Machine, RF; Random Forest, GNB; Gaussian Naive Bayes, LDA; Linear Discriminant Analysis KNN; K-Nearest Neighbors, GBM; Gradient Boosting Machine, XGB; Extreme Gradient Boosting, CAT; Cat Boost, LGBM; Light Gradient Boosting Machine. (DOCX) [file pone.0300201.s002.docx]

**Supplementary Table 2.** Finding the appropriate hyper-parameter values for each algorithm after hyper-parameter tuning

| Algorithm | Best hyper-parameters |
| --- | --- |
| LR | C=10, solver='lbfgs', multiclass='auto' |
| SVM | C = 1, gamma = 'scale', kernel= 'rbf', probability=True |
| RF | max_features = 'sqrt', n_estimators = 1000 |
| GNB | var_smoothing = 0.01 |
| LDA | shrinkage= 'auto', solver= 'lsqr', tol= 0.0001 |
| KNN | metric= 'euclidean', n_neighbors= 13, weights= 'uniform' |
| GBM | learning_rate= 0.01, max_depth=9, n_estimators= 1000, subsample= 0.5 |
| XGB | n_estimators=180, colsample_bytree= 0.5, gamma= 1, max_depth=1 , min_child_weight=10, reg_alpha=1, reg_lambda=1 |
| CAT | loss_function='Logloss',iterations=120,verbose=False,depth=4,learning_rate=0.04 |
| LGBM | n_estimators=200,learning_rate=0.01,num_leaves=40,extra_trees=True,drop_rate=0.1 |
| #Abbreviations, LR; Logistic Regression, SVM; Support Vector Machine, RF; Random Forest, GNB; Gaussian Naive Bayes, LDA; Linear Discriminant Analysis KNN; K-Nearest Neighbors, GBM; Gradient Boosting Machine, XGB; Extreme Gradient Boosting, CAT; Cat Boost, LGBM; Light Gradient Boosting Machine | |
