# Supplementary material for: Machine learning-based models to predict the conversion of normal blood pressure to hypertension within 5-year follow-up
Source: PLoS One. 2024 Mar 14;19(3):e0300201. doi: 10.1371/journal.pone.0300201 (PMC10939282; doi:10.1371/journal.pone.0300201)
Supplement: S3 Table — #Abbreviations, LR; Logistic Regression, SVM; Support Vector Machine, RF; Random Forest, GNB; Gaussian Naive Bayes, LDA; Linear Discriminant Analysis, KNN; K-Nearest Neighbors, GBM; Gradient Boosting Machine, XGB; Extreme Gradient Boosting, CAT; Cat boost, LGBM; Light Gradient Boosting Machine, AUC; Area Under the ROC Curve, ROC; Receiver operating characteristic, AUC-PR; Area Under the Precision-Recall curve. (DOCX) [file pone.0300201.s003.docx]

**Supplementary Table 3.** Performance of the ten machine learning algorithms using all features

| Algorithm | Accuracy | sensitivity | specificity | F1 score | AUC | AUC-PR |
| --- | --- | --- | --- | --- | --- | --- |
| LR | 0.85  (0.78-0.92) | 0.14  (0.1-0.18) | 0.94  (0.89-0.99) | 0.17  (0.12-0.22) | 0.58  (0.49-0.67) | 0.16  (0.12-0.21) |
| SVM | 0.46  (0.38-0.54) | 0.62  (0.53-0.71) | 0.44  (0.36-0.52) | 0.20  (0.15-0.25) | 0.56  (0.48-0.65) | 0.11  (0.08-0.15) |
| RF | 0.88  (0.82-0.94) | 0.08  (0.05-0.11) | 0.98  (0.95-1.0) | 0.12  (0.08-0.16) | 0.65  (0.56-0.74) | 0.19  (0.14-0.24) |
| GNB | 0.34  (0.27-0.41) | 0.82  (0.75-0.89) | 0.29  (0.22-0.36) | 0.21  (0.16-0.26) | 0.58  (0.50-0.67) | 0.13  (0.09-0.17) |
| LDA | 0.77  (0.69-0.85) | 0.24  (0.18-0.30) | 0.84  (0.77-0.91) | 0.19  (0.14-0.24) | 0.57  (0.49-0.66) | 0.14  (0.10-0.18) |
| KNN | 0.51  (0.42-0.60) | 0.44  (0.36-0.52) | 0.52  (0.43-0.61) | 0.16  (0.12-0.20) | 0.48  (0.39-0.56) | 0.09  (0.06-0.12) |
| GBM | 0.88  (0.82-0.94) | 0.08  (0.05-0.11) | 0.98  (0.95-1.0) | 0.13  (0.09-0.17) | 0.63  (0.55-0.72) | 0.16  (0.12-0.21) |
| XGB | 0.81  (0.74-0.88) | 0.20  (0.15-0.25) | 0.89  (0.83-0.95) | 0.19  (0.14-0.24) | 0.58  (0.49-0.66) | 0.15  (0.11-0.20) |
| CAT | 0.83  (0.76-0.90) | 0.26  (0.20-0.32) | 0.90  (0.84-0.96) | 0.25  (0.19-0.31) | 0.59  (0.51-0.68) | 0.17  (0.12-0.21) |
| LGBM | 0.82  (0.75-0.89) | 0.18  (0.13-0.23) | 0.90  (0.84-0.96) | 0.19  (0.14-0.24) | 0.63  (0.54-0.71) | 0.20  (0.15-0.25) |

**#Abbreviations**, **LR**; Logistic Regression, **SVM**; Support Vector Machine, **RF**; Random Forest, **GNB**; Gaussian Naive Bayes, **LDA**; Linear Discriminant Analysis, **KNN**; K-Nearest Neighbors, **GBM**; Gradient Boosting Machine, **XGB**; Extreme Gradient Boosting, **CAT**; Cat boost, **LGBM**; Light Gradient Boosting Machine, **AUC**; Area Under the ROC Curve, **ROC**; Receiver operating characteristic, **AUC-PR**; Area Under the Precision-Recall curve.
