# Supplementary material for: Machine learning-based models to predict the conversion of normal blood pressure to hypertension within 5-year follow-up
Source: PLoS One. 2024 Mar 14;19(3):e0300201. doi: 10.1371/journal.pone.0300201 (PMC10939282; doi:10.1371/journal.pone.0300201)
Supplement: S4 Table — #Abbreviations, LGBM; Light Gradient Boosting Machine, AUC; Area Under the ROC Curve, ROC; Receiver operating characteristic, AUC-PR; Area Under the Precision-Recall curve. (DOCX) [file pone.0300201.s004.docx]

**Supplementary Table 4.** Performance of the LGBM model with different number of features

| Algorithm | Accuracy | sensitivity | specificity | F1 score | AUC | AUC-PR |
| --- | --- | --- | --- | --- | --- | --- |
| Top-10 features | 0.73  (0.65-0.81) | 0.44  (0.36-0.52) | 0.76  (0.68-0.84) | 0.24  (0.18-0.30) | 0.66  (0.57-0.74) | 0.18  (0.12-0.24) |
| Top-15 features | 0.76  (0.68-0.84) | 0.36  (0.29-0.43) | 0.81  (0.74-0.88) | 0.18  (0.12-0.24) | 0.64  (0.55-0.72) | 0.17  (0.11-0.23) |
| Top-20 features | 0.76  (0.68-0.84) | 0.28  (0.22-0.34) | 0.82  (0.75-0.89) | 0.22  (0.17-0.27) | 0.64  (0.55-0.73) | 0.19  (0.13-0.25) |
| Top-25 features | 0.78  (0.7-0.86) | 0.34  (0.27-0.41) | 0.83  (0.76-0.9) | 0.22  (0.16-0.28) | 0.64  (0.56-0.73) | 0.19  (0.13-0.25) |
| Top-30 features | 0.80  (0.72-0.88) | 0.34  (0.27-0.41) | 0.85  (0.78-0.92) | 0.23  (0.27-0.29) | 0.67  (0.59-0.76) | 0.26  (0.20-0.31) |
| Top-35 features | 0.80  (0.72-0.88) | 0.32  (0.25-0.39) | 0.86  (0.79-0.93) | 0.22  (0.18-0.28) | 0.67  (0.59-0.76) | 0.26  (0.20-0.31) |
| all features | 0.82  (0.75-0.89) | 0.18  (0.13-0.23) | 0.90  (0.84-0.96) | 0.19  (0.14-0.24) | 0.63  (0.54-0.71) | 0.18  (0.13-0.23) |

**#Abbreviations**, **LGBM**; Light Gradient Boosting Machine, **AUC**; Area Under the ROC Curve, **ROC**; Receiver operating characteristic, **AUC-PR**; Area Under the Precision-Recall curve.
